# Medical student attitudes towards older people: a critical review of quantitative measures

**DOI:** 10.1186/s13104-018-3186-z

**Published:** 2018-01-24

**Authors:** Mark A. G. Wilson, Susan Kurrle, Ian Wilson

**Affiliations:** 1 Graduate Medicine, School of Medicine, University of Wollongong, PO Box 1782, Bowral, NSW 2576 Australia; 2 Health Care of Older People, Faculty of Medicine, University of Sydney, Hornsby Ku-ring-gai Health Service, Hornsby, NSW 2077 Australia; 3 Learning and Teaching, School of Medicine, Faculty of Science, Medicine and Health, University of Wollongong, Building 28 Rm 115, Wollongong, NSW 2522 Australia

**Keywords:** Instrument, Quantify, Medical student, Attitudes, Aged persons

## Abstract

**Objectives:**

Further research into medical student attitudes towards older people is important, and requires accurate and detailed evaluative methodology. The two objectives for this paper are: (1) From the literature, to critically review instruments of measure for medical student attitudes towards older people, and (2) To recommend the most appropriate quantitative instrument for future research into medical student attitudes towards older people.

**Results:**

A SCOPUS and Ovid cross search was performed using the keywords Attitude and medical student and aged or older or elderly. This search was supplemented by manual searching, guided by citations in articles identified by the initial literature search, using the SCOPUS and PubMed databases. International studies quantifying medical student attitudes have demonstrated neutral to positive attitudes towards older people, using various instruments. The most commonly used instruments are the Ageing Semantic Differential (ASD) and the University of California Los Angeles Geriatric Attitudes Scale, with several other measures occasionally used. All instruments used to date have inherent weaknesses. A reliable and valid instrument with which to quantify modern medical student attitudes towards older people has not yet been developed. Adaptation of the ASD for contemporary usage is recommended.

## Introduction

Medical student attitudes towards older people in the community are important to understand and quantify. Ageist attitudes, ubiquitous in the healthcare sector, may influence medical practice [1]. There are many examples of ageism in the literature, including the reticence of some primary care physicians to take on the care of older people [2], provision of less information to older people by doctors [3], cardiologists offering a narrower range of options to older patients [4], and specialists offering less aggressive treatment to older women with breast cancer [5]. To ameliorate such ageism, fostering development of positive attitudes towards older people during medical training must be a fundamental outcome of medical curricula.

Fixed medical student views about older people have been studied for over 50 years. The first longitudinal study investigating attitudes recently provided evidence that student attitudes towards older people decline throughout medical school [6]. As attitudes are complex and multi-dimensional, both qualitative and quantitative research are required to provide more comprehensive understanding. One of the greatest challenges in understanding student attitudes has been the plethora of instruments used in their measurement, each with particular deficiencies. The aims of this paper are to critically review the instruments which have been utilised to quantify medical student attitudes, and to identify the optimal instrument type for future medical education research.

## Main text

### Method

On 18th March 2016, 171 articles were identified utilising the following keywords: Attitudes AND medical student AND older OR old OR elderly in the database SCOPUS, and by manual searching directed by article citations, using SCOPUS and PubMed. An additional 371 articles were found by an Ovid Cross Search using the same search parameters. During March and April 2016, from a total of 542 articles identified by the search, 299 were found to be relevant, after eliminating those not in English (29), duplicated (147), or found to be unrelated to the area of interest by screening title, abstract and text (76).

**Fig. 1:**
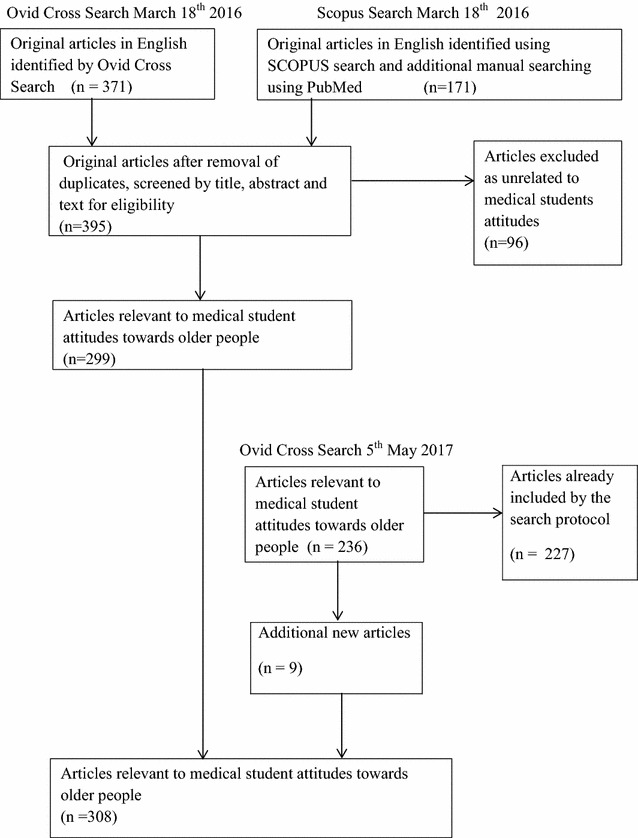
Search strategy for literature review of attitudes of medical students towards older people. A total of 308 peer-reviewed journal articles were reviewed to inform this paper. In March 2016, 542 articles were identified utilising the keywords **attitudes** and **medical student** and **older** or **old** or **elderly** in the database SCOPUS, by manual searching directed by article citations, using SCOPUS and PubMed, and by an Ovid Cross Search using the same search parameters. 299 articles were found to be relevant, after eliminating those not in English, duplicated, or unrelated to the area of interest by screening title, abstract and text. An additional Ovid Cross Search, performed using the same search terms in May 2017, identified 9 new articles since the original search

An additional Ovid Cross Search was performed using the same search terms on May 5 2017, identifying 9 new articles since the original search. A total of 308 peer-reviewed journal articles were thus reviewed to inform this paper (see Fig. 1).

### Results

Three systematic reviews of health professional, including medical student, attitudes towards older adults were identified from the literature [7–9]. In addition to these studies, several other studies of medical student attitudes were identified by this review. Most research has quantified the effect of geriatric medical curriculum innovation on medical student attitudes, and has either shown neutral or positive effect. Some studies simply sought to describe the attitudes of a cohort of medical students. Thirty- one relevant studies, including instruments used to measure attitudes, are summarised in Table 1.

**Table 1:** Quantitative studies investigating medical student attitudes towards older people

Author	Study description	Quantitative measure of attitudes utilised	Study finding
Beer et al. (2011) [10]	Cross sectional survey of students from two medical schools (1 undergraduate and 1 postgraduate course) and geriatric medicine teachers: Response rate for students 14% (208/760). Australia	UCLA-GAS	Responses of students and teachers generally similar. Teachers had more positive responses to the first 4 items of the scale
Bernard et al. (2003) [11] ]	Non- randomised trial: 225 first and second year medical students. Intervention-healthy senior mentorship program over 2 years vs control group. USA	ASD	Attitudes improved in both cohorts, significantly more in the intervention group
Brand et al. (2016) [12]	Mixed methodology. Qualitative plus pre and post–test: 128 first year medical students. Intervention-curriculum innovation. Australia	Modified UCLA-GAS	Positive attitude change in 8 of 13 test items
Cheong et al. (2009) [13]	Cross sectional survey: 218 first year and 124 third year medical students. Singapore	Kogan’s Attitude To Old Person Scale (KOAP)	Positive mean attitudes
Chua et al. (2008) [14]	Cross sectional survey: 250 first year medical students at admission. Singapore	Modified UCLA-GAS	Positive mean attitudes score
Diachun et al. (2010) [15]	Randomised controlled study: 196 (75% response rate) third year medical students recruited. Intervention-2 week geriatric rotation vs non-geriatric rotation. Canada	Modified UCLA-GAS	The attitudes of the intervention group did not deteriorate as much as the control group
Duke et al. (2009) [16]	Pre and post- test study: 71 first year medical students. Intervention-senior mentoring program. USA	Modified UCLA-GAS	Positive change in attitudes measured
Eskildsen & Flacker (2009) [17]	Pre and post-test study: 129 first year medical students. Intervention-short geriatric course. USA.	UCLA-GAS	Positive change in attitudes measured
Fields et al. (1992) [18]	Pre and post-test study: 127 fourth year medical students. Intervention-geriatrics rotation. USA	ASD	No measurable change in attitudes
Gonzales et al. (2010) [19]	Cross sectional validity study: 199 first year (91%) and second year (9%) medical students recruited voluntarily. USA	Polizzi’s refined ASD	Validity of the instrument could not be confirmed by structural equation modelling
Hall et al. (1997) [20]	Controlled trial, pre and post–test: 162 (63% response rate) fourth year medical students recruited. Intervention short geriatric course. USA	Modified Maxwell- Sullivan Attitudes Scale (MSAS)	No measurable change in attitudes
Hughes et al. (2008) [21]	Cross sectional survey of students (first year), pre and post–test study(fourth year): 165 first years (99% response rate) and 70 (58% response rate) fourth year medical students. Intervention -geriatric short course in fourth year. UK	Modified UCLA-GAS	More positive attitudes in fourth year students compared with first year students. No measurable change in attitudes after the intervention
Intrieri et al. (1993) [22]	Pre and post-test study with comparison group: 96 third year medical students. Psychiatry clinical rotation with gerontology (intervention) vs psychiatry alone (comparison group) USA	ASD	Positive change in attitudes
Koh et al. (2012) [23]	Pre and post-test, with control: Intervention group (261) second year students, holistic curriculum in geriatric medicine. Control group (254). Singapore	Modified UCLA-GAS	Positive change in attitudes
Lorraine et al. (1998) [24]	Pre and post-test study: 100 fourth year medical students. Intervention-Brief “ageing simulation” intervention. USA	ASD	Positive change in attitudes
Lu et al. (2010) [25]	Pre and post- test study with comparison group: 137 (71% response rate) first year medical students. Intervention-Healthy senior mentorship. USA	ASD	No change in attitudes
Muangpaisan, Intalapapron & Assantachai (2008) [26]	Cross sectional survey: 146 fourth year medical students (Response rate 61%) and 60 medical residents (Response rate 50%). Thailand	UCLA-GAS	Attitudes positive, no significant difference between students and graduates
Nagoshi et al. (2008) [27]	Cohort study with comparison group: 59 medical students surveyed at beginning and end of course. Intervention-new course curriculum. USA	UCLA-GAS	No difference in attitudes between groups
Pacala et al. (1995) [28]	Pre and post-test study with comparison group: 55 fourth year medical students. Intervention-ageing simulation workshop. USA	ASD, modified MSAS.	Positive change in attitudes.
Roscoe et al. (2005) [29]	Pre and post-test study: 252 third year medical students (89% response rate). Intervention-short geriatrics course. USA	Modified ASD	Positive change in attitudes
Sahin et al. (2012) [30]	Cross sectional survey: 106 health professional students, including 43 medical students, and 150 postgraduates. Turkey	Modified UCLA-GAS	Doctors had more positive attitudes than students.
Seaman et al. (2017) [31]	Pre and post- test study: 51 volunteer health professional students, including a small number of medical students. Intervention-Inter-professional team work in an aged care facility. Australia	ASD	Positive change in attitudes, but numbers too small for significance
Shue et al. (2005) [32]	Pre and post-test study with comparison group: 161 first year medical students. Intervention-senior mentorship program. USA	ASD, modified MSAS	Positive change in attitudes
Stewart et al. (2007) [33]	Non-randomised controlled trial: Four sequential cohorts of 249 medical students. Intervention-new geriatric curriculum across course. USA	ASD	Neutral attitudes, no measurable difference between cohort attitudes
Tam et al. (2014) [34]	Pre and post-test study: 60 (82% response rate) fifth year medical students. Intervention-curriculum change. Australia	UCLA-GAS	Positive change in attitudes
Ten Haken et al. (1995) [35]	Pre and post-test longitudinal study: 117 (63% response rate) undergraduate medical students. Intervention-clinical skills course. USA	Modified ASD	No sustained change in attitudes
Varkey et al. (2006) [36]	Pre and post-test study: 84 first year medical students. Intervention-ageing game ‘one off’ intervention. USA	MSAS, ASD	Positive change in attitudes
Watson (2013) [37]	Cross sectional survey: 129 third to fifth year medical students. Australia	UCLA-GAS	Neutral to positive across scales
Wilkinson, Gower, Sainsbury (2002) [38]	Pre and post-test study with comparison group: 186 second year students had intervention. 62 of this cohort were followed up in fourth year, compared with 160 controls. Intervention-community contact in second year and 4 week attachment in 4th year. New Zealand	ASD	Positive change in attitudes measured in second year and fourth year
Wilson & Gamser (1982) [39]	Pre and post-test study: 61 first year medical students (Response rate 74%). Intervention-short geriatrics course. USA	ASD	Positive change in attitudes
Zwahlen et al. (2010) [40]	Pre and post-test study: 347 (Response rate 81%) undergraduate medical students across the medical course. Intervention-New course curriculum. USA	UCLA-GAS	No change in attitudes

*ASD* Ageing Semantic Differential, *UCLA*-*GAS* University of California Los Angeles Geriatric Attitude Scale, *KAOP* Kogan’s Attitude to Old Person, *MSAS* Maxwell-Sullivan Attitudes Survey

#### A description and comparison of the instruments used for measurement of medical student attitudes towards older people

##### The Ageing Semantic Differential (ASD)

The most widely used instrument in published studies of medical student attitudes towards older people has been the ASD. The construct of semantic differential was first adapted to study social stereotypes in 1946 [41], introducing the potential for this instrument type to test multiple dimensions of attitudes [42]. The ASD directs respondents to indicate which of thirty-two polar adjectives best describes their attitude to an older person across a seven step scale. The subject is asked to indicate the point on the scale which represents the direction and intensity of his or her judgement between each pair of polar opposite adjectives. Three major dimensions were identified by factor analysis: instrumental-ineffective, autonomous-dependent, and personal acceptability-unacceptability [43].

The semantic differential (SD) approach to quantifying medical student attitudes has several strengths: SD eliminates the problem of statements within instruments, which may capture beliefs rather than attitudes, a potential flaw of many instruments [44]. The ASD more specifically quantifies attitudes, whereas other instruments such as the Kogan Attitude Toward Old Persons Scale [45] or the Palmore scale [46] confound attitudes with knowledge. SD requires relatively short survey times for measuring complex concepts [47], and has reported superior reliability and validity over Likert-based or Stapel scales [48].

While the ASD is widely used, there are three main areas of potential weakness:

1. Many adjectives employed by the ASD are outdated, with polar adjectives derived from surveys done in the United States of America in the 1950s [43]. Selection criteria of words for the scale are unclear. The pilot study tested the instrument on non-medical undergraduates at the University of Missouri. Some argue that vague or unfamiliar adjectives may result in students choosing more neutral responses [33].
2. The original work did not evaluate young people’s attitudes towards older women. The original factor analysis of the ASD asked respondents to use the ASD to evaluate three different age groups of men, the oldest attitudinal object group consisting of men 70 to 85 years of age [43]. Sexism has no place today in the accurate measurement of attitudes towards older people.
3. There is a question of whether the ASD has validity, and whether it measures what it was intended to measure. The factor structure of the original ASD has been debated [49, 50], with some favouring a four factor structure as a better fit to the data [44].

##### Polizzi’s refined ageing semantic differential

The original ASD was refined in 2003, converting the instrument to 24 adjectival pairs using only one factor, the evaluative factor [50]. Polizzi’s refined ASD has been criticised as a having poor fit using structural equation modelling [51]. A recent US study evaluating the validity of the refined ASD concluded that the refined ASD lacked validity, and was a unidimensional instrument. On the basis of qualitative data from this study, a four factor instrument was postulated, with experience the new factor [19].

##### The University of California Los Angeles Geriatric Attitude Scale (UCLA-GAS) and modified versions

Another widely used instrument to measure attitudes of medical students towards older people is the UCLA-GAS. The UCLA-GAS is a 14 item survey using Likert-scale responses indicating whether the respondent agrees or disagrees with the statement. Cronbach’s alpha in the original work was 0.76, with good construct validity [52]. However, the UCLA-GAS makes use of five positive and nine negative statements about older patients, exposing the method to criticisms including the tool measures beliefs rather than attitudes [44], and is unbalanced or may have other problems with construct validity [53]. Some authors have expressed a view that the UCLA-GAS by its very structure may inadvertently support the messages of ageism [16]. Initially developed for medical residents in 1998 [52], the UCLA-GAS has been used to investigate medical student attitudes. In US studies, the internal reliability for the UCLA-GAS or modified version has been sub-optimal (Cronbach’s alpha 0.69) in studies outside UCLA [53–55].

Despite concerns regarding the reliability and validity in measuring medical student attitudes in the US, the UCLA-GAS has been used internationally, often with modification. Three items were modified in the Singapore UCLA-GAS, with Cronbach’s alpha 0.73 when administered to first year medical students [14], but alpha of 0.61 -0.69 in a subsequent Singapore study [23]. Turkish investigators studied medical student attitudes using a literally translated UCLA-GAS instrument. Cronbach’s alpha was 0.67. An attempt was made to show validity by comparing student responses on a local unvalidated scale of elderly discrimination attitudes [30]. The authors of a study comparing attitudes of medical students and residents towards older people in Thailand used a modified UCLA-GAS, finding no significant difference between student and resident attitudes, not describing reliability [26]. One Australian study of fourth year medical students and their teachers provides evidence of internal reliability (Cronbach’s alpha 0.78) for the UCLA-GAS and some evidence of content validity, with geriatric medicine teachers having more positive attitudes scores than their students [10].

##### The Maxwell-Sullivan Attitudes Survey (MSAS)

Another instrument occasionally used to quantify attitudes towards older patients is the MSAS [56], developed for use with trainees in family medicine [52]. It is a 28 item survey within five scales, in part attitudinal, but also concerning educational preparedness to manage older patients. Significant concerns about the reliability and validity of the MSAS [33, 52] limit the scale’s utility in medical student attitudes research.

##### Kogan’s Attitude to Old Person Scale

As indicated earlier, this scale confounds knowledge and beliefs with attitudes, and has seldom been used to measure medical student attitudes. There are flaws in the structure of this scale, making its psychometric utility questionable [51].

#### Other quantitative instruments for measuring medical student attitudes to older people

##### Fraboni’s Scale on Ageism

Fraboni’s Scale on Ageism (FSA), developed from studies of Canadian high school and college students, and workers, consists of 29 statements which evaluate attitudes towards older people [57]. In studies of age bias in university students, the FSA has some evidence of reliability and validity [58]. The scale has scarcely been used to investigate medical professional attitudes. Used in one Australian study of hospital doctors’ attitudes, results indicated neutral to positive mean attitudes [59]. Validity and reliability of the scale for either medical graduate or student research is unknown.

##### Carolina opinions on care of older adults

A more recently developed instrument, the Carolina Opinions on Care of Older Adults, was developed in view of questions in relation to the reliability and generalizability of other instruments [60]. While promising in a North Carolina context, there is no further published evidence of repeatability and reliability for this instrument.

##### Implicit association test

Another interesting area of research with regard to student attitudes towards older people are implicit attitude measures. The Implicit Association Test involves a rapid sorting task between two contrasting categories, comparing response latencies to stimuli, for example adjectives or faces of younger versus older people [61]. Whilst not described in medical student research, a study of psychology students demonstrated positive explicit attitudes but neutral implicit attitudes toward older people [61]. The authors postulate that respondents may avoid a socially undesirable response to explicit attitudes instruments.

### Discussion

Currently no reliable and well validated instrument is available for use in quantitative research into medical student attitudes towards older people. On reviewing the literature where instruments have been used to quantify medical student attitudes towards older people, the instrument type with the most positive attributes is semantic differential (SD). A SD has the potential to reliably measure complex attitudes in a short space of time [47], which is important when surveying busy medical students. A well- constructed SD instrument should be more specific in measurement of student attitudes than explicit instruments utilising statements that may confound student attitudes with beliefs and/or knowledge about older people [44, 51]. Respondents’ evaluative responses are less likely to be inhibited by an SD as compared with more explicit instruments [61]. Within a semantic differential, several dimensions of medical student attitudes may be evaluated, potentially providing greater insights.

Although the ASD has been extensively used for research of medical student attitudes toward older people, important flaws in this tool require addressing. The adjectives in a refined ASD instrument should reflect contemporary language, the evaluation should be of an older male or female person, and the newly refined instrument should undergo appropriate tests for reliability and validity in the context where it is utilised. A previous attempt made by Polizzi to refine the ASD [50], has proven to be inadequate [19, 51].

Medical student attitudes towards older people must be accurately quantified and understood for medical educators to effectively develop curricula in geriatric medicine which foster positive attitudes to older people as a core graduate outcome. We suggest that a properly validated modified ASD instrument be developed, using contemporary language and designed to measure multiple dimensions of medical student attitudes towards older people. Future quantitative studies should be complemented by qualitative data to more fully inform educators in geriatric medicine.

## Limitations

It is possible that a systematic review may have identified additional instruments or evidence to inform this critical review. Attitudes are complex, multi-dimensional and challenging to accurately quantify in medical education research.

## Abbreviations

ASD: Ageing Semantic Differential
UCLA-GAS: University of California Los Angeles Geriatric Attitude Scale
KAOP: Kogan’s Attitude to Old Person
SD: semantic differential
MSAS: Maxwell-Sullivan Attitudes Survey
FSA: Fraboni’s Scale on Ageism
